# Surgical outcomes of robotic-assisted percutaneous fixation for thoracolumbar fractures in patients with ankylosing spondylitis

**DOI:** 10.1186/s12891-024-07597-6

**Published:** 2024-06-20

**Authors:** Zhi-Yuan Ye, Jin-Yu Bai, Zhi-Min Ye, Xu-Shen Zhao, Fang-Long Song, Zhen-Tao Zhou, Bing-Chen Shan, Xiao-Zhong Zhou

**Affiliations:** 1 Department of Orthopedics, The Second Affiliated Hospital of Soochow University, No. 1055, Sanxiang Road, Suzhou, 215004 China; 2 Department of Orthopedics, Sihong Hospital, Sihong, Jiangsu China; 3 Department of Image, Sihong Geriatric Hospital, Sihong, Jiangsu China

**Keywords:** Ankylosing spondylitis, Thoracolumbar fractures, Surgical outcomes, Percutaneous techniques, Robot

## Abstract

**Background:**

Spinal fractures in patients with ankylosing spondylitis (AS) mainly present as instability, involving all three columns of the spine, and surgical intervention is often considered necessary. However, in AS patients, the significant alterations in bony structure and anatomy result in a lack of identifiable landmarks, which increases the difficulty of pedicle screw implantation. Therefore, we present the clinical outcomes of robotic-assisted percutaneous fixation for thoracolumbar fractures in patients with AS.

**Methods:**

A retrospective review was conducted on a series of 12 patients diagnosed with AS. All patients sustained thoracolumbar fractures between October 2018 and October 2022 and underwent posterior robotic-assisted percutaneous fixation procedures. Outcomes of interest included operative time, intra-operative blood loss, complications, duration of hospital stay and fracture union. The clinical outcomes were assessed using the visual analogue scale (VAS) and Oswestry Disability Index (ODI). To investigate the achieved operative correction, pre- and postoperative radiographs in the lateral plane were analyzed by measuring the Cobb angle.

**Results:**

The 12 patients had a mean age of 62.8 ± 13.0 years and a mean follow-up duration of 32.7 ± 18.9 months. Mean hospital stay duration was 15 ± 8.0 days. The mean operative time was 119.6 ± 32.2 min, and the median blood loss was 50 (50, 250) ml. The VAS value improved from 6.8 ± 0.9 preoperatively to 1.3 ± 1.0 at the final follow-up (*P* < 0.05). The ODI value improved from 83.6 ± 6.1% preoperatively to 11.8 ± 6.6% at the latest follow-up (*P* < 0.05). The average Cobb angle changed from 15.2 ± 11.0 pre-operatively to 8.3 ± 7.1 at final follow-up (*P* < 0.05). Bone healing was consistently achieved, with an average healing time of 6 (5.3, 7.0) months. Of the 108 screws implanted, 2 (1.9%) were improperly positioned. One patient experienced delayed nerve injury after the operation, but the nerve function returned to normal upon discharge.

**Conclusion:**

Posterior robotic-assisted percutaneous internal fixation can be used as an ideal surgical treatment for thoracolumbar fractures in AS patients. However, while robot-assisted pedicle screw placement can enhance the accuracy of pedicle screw insertion, it should not be relied upon solely.

## Background

Ankylosing Spondylitis (AS) is a chronic inflammatory disease which is characterized by pain and progressive stiffness [1]. Considering the unique characteristics of spinal fusion, osteoporosis, and spinal deformities in AS patients, individuals affected by these conditions are more prone to experiencing fractures even with minimal force impact [2–4]. The prevalence of spinal fractures in patients with AS is believed to be four times higher than that in healthy individuals [5]. Because these fractures often occur after minor trauma and in individuals with pre-existing chronic back pain, reaching a diagnosis is often challenging and may result in secondary neurological deficits [2].

The spinal fractures in patients with AS are predominantly unstable, involving all three columns of the spine, which require effective treatment in the early stage [5]. Due to the prevalence of unstable injuries and a higher occurrence of neurological symptoms, surgical intervention is often considered essential [6, 7]. The traditional posterior open surgery is the classic treatment for AS, which achieves good clinical results [8, 9]. However, due to the inflammatory reaction and osteoporosis of the disease itself, extensive dissection of paraspinal muscles during the operation will leads to increased bleeding, prolonged operation time, and an increased risk of postoperative infection [9].

In recent years, there has been an increasing inclination towards the utilization of percutaneous techniques for spinal fracture instrumentation. This approach offers advantages such as reduced surgical duration, minimal blood loss, and shorter hospital stays for individuals with AS who have thoracolumbar fractures [9–12]. However, in patients with AS, the significant alterations in bony structure and anatomy result in a lack of identifiable landmarks, which increases the difficulty of pedicle screw implantation and imposes demanding technical requirements on the operator [9]. Therefore, robot-assisted surgical fixation for AS combined with thoracolumbar fractures has become possible, because it has been confirmed to have the advantages of minimizing radiation exposure and improving the accuracy of screw placement in the treatment of common spinal fractures [13–15]. In addition, by utilizing robotic assistance, surgeons have the flexibility to select screws with a larger diameter and increased length [16]. This theoretically provides a more stable healing environment for thoracolumbar fractures in patients with AS.

Here, we present a study on patients with AS who underwent robotic-assisted percutaneous fixation for thoracolumbar fractures. To the best of our knowledge, this is the first case series investigating the clinical effect of robotic-assisted percutaneous fixation in treating thoracolumbar fractures in AS patients, which may provide a new treatment option. We thoroughly analyze our results, focusing on outcomes and complications, and compare them to previously published data.

## Materials and methods

### Patients

This study was approved by the Institutional Review Board of the Second Affiliated Hospital of Soochow University, and all patients signed informed consent forms. A retrospective review was conducted on all patients diagnosed with thoracolumbar fractures in AS who underwent robotic-assisted percutaneous fixation at our institution from October 2018 to October 2022. All patients received plain radiography, computed tomography (CT), magnetic resonance imaging, and a physical examination by a spinal surgeon upon their admission to the hospital. Patients who had a delay of over 24 h in receiving a diagnosis for their fractures were classified as having experienced a delayed identification. Diagnosis was made based on clinical and radiographic assessment. The AO Spine Thoracolumbar Spine Injury Classification System was utilized for the categorization of spinal fractures. Neurological impairments were evaluated using the American Spinal Injury Association (ASIA) grading system. Age, gender, trauma history, fracture level, delayed diagnosis, duration between diagnosis and operation, body mass index and C-reactive protein levels were recorded (Table 1).

**Table 1:** Patient demographics and case details

Case	Gender (F/M)	Age (years)	Trauma history	Fracture level	AO classification (fracture type)	ASIA grade preoperatively	Delayed diagnosis	Days between diagnosis and operation	CRP (mg/L)	BMI (kg/m^2^)
1	F	65	No	L2	B2	ASIA E	Yes	3	6.1	32.87
2	M	57	Fall from ladder (high impact)	T5	B2	ASIA E	NO	20	171.2	24.80
3	M	48	No	L1, L2	B2	ASIA D	Yes	4	25.8	23.53
4	M	83	Fall from standing (low impact)	L3	B3	ASIA E	Yes	5	100.1	25.39
5	M	68	No	T10、T11	B3	ASIA E	Yes	6	51.6	27.24
6	M	69	Fall from standing (low impact)	T12	B3	ASIA E	Yes	3	57.4	25.95
7	M	79	Fall from standing (low impact)	T12	B3	ASIA E	NO	3	60.9	26.81
8	M	66	No	L1, L2	B3	ASIA E	Yes	1	7.0	25.71
9	M	49	Fall from ladder (high impact)	T10	B3	ASIA E	Yes	4	7.3	23.74
10	M	41	Fall from standing (low impact)	L2	B3	ASIA E	NO	4	110.5	24.8
11	M	54	Fall from standing (low impact)	L2	B3	ASIA E	NO	5	98.6	20.3
12	M	74	Fall from standing (low impact)	T11	B3	ASIA E	No	4	43.3	19.59

F, female; M, male; L, lumbar; T, thoracic; BMI, body mass index; CRP, C-reactive protein; ASIA, American Spinal Injury Association

### Inclusion and exclusion criteria

The inclusion criteria were as follows: (1) AS was diagnosed based on the modified New York criteria; (2) the imaging findings were thoracolumbar fracture; (3) the patient underwent posterior robotic-assisted percutaneous fixation for treatment. The exclusion criteria were as follows: (1) The patient was treated conservatively or with other procedures without robotic assistance; (2) the patient has thoracolumbar fracture combined with multiple concomitant fractures; (3) the patient is unable to tolerate surgery for personal reasons; (4) the incompleteness of radiological information, treatment details, and follow-up data.

### Surgical technique

**Fig. 1:**
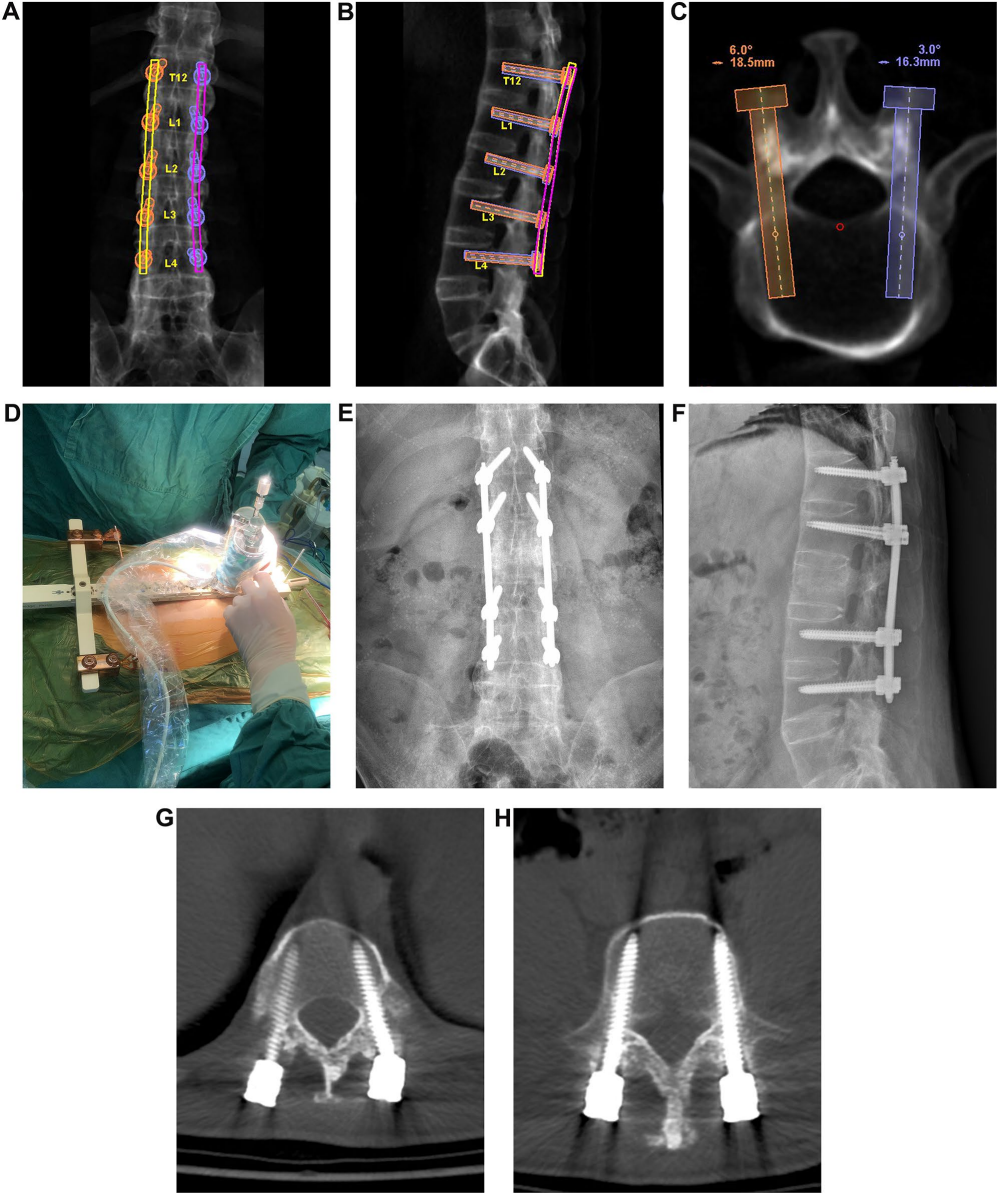
A 41-year-old man, who was diagnosed with an L2 fracture, had a history of low-impact trauma. (**A**-**C**) The preoperative planning involves determining the optimal trajectory for screw placement and selecting the appropriate size of pedicle screw. (**D**) The robotic manipulator was positioned on the bone-mounted platform, and the appropriate pedicle screws were inserted. Postoperative anteroposterior (**E**) and lateral radiographs (**F**), as well as transverse computed tomography scans (**G**-**H**), demonstrate satisfactory screw placement and sizing

**Fig. 2:**
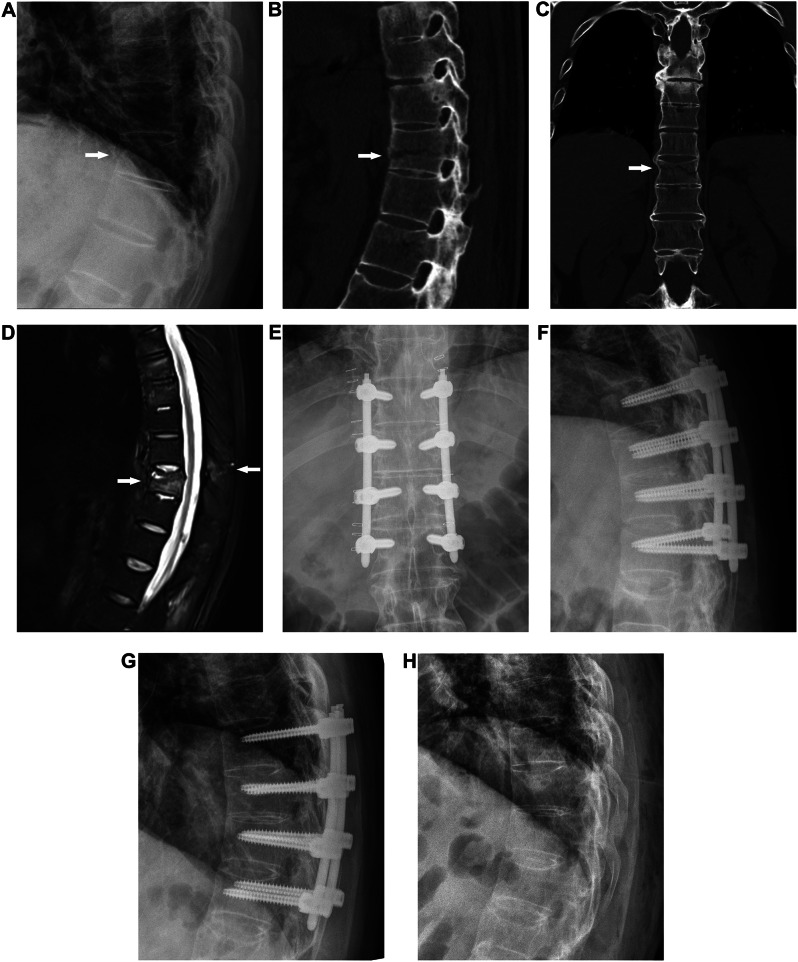
A 51-year-old man was diagnosed with a T11 fracture after sustaining an injury from a fall. Lateral radiography (**A**) reveals morphological changes in the T11 vertebral body (arrow). Preoperative computed tomography (**B**-**C**) demonstrated a fracture traversing the T11 vertebral body, pedicle, and articular process (arrow). Preoperative magnetic resonance imaging (**D**) showing a 3-column injury at T11 (arrow). Postoperative plain radiographs (**E**-**F**) demonstrate satisfactory screw placement. Lateral radiography (**G**-**H**) showed that the fracture had healed two years after the operation, and the internal fixation had been removed

All surgical procedures were performed on a specially designed flexible operating bed with the patient in a prone position and under general anesthesia. Sufficient cushioning was applied to accommodate the kyphotic deformity and minimize the risk of spinal cord injury. The CT scan of the surgical area was sent to the workstation prior to the operation (Renaissance; Mazor Robotics Ltd., Caesarea, Israel). The surgeon’s requests for precise vertebral trajectories and screw dimensions were meticulously planned one day prior to the surgery (Fig. 1A-C). During the preparation surgery, registration was performed using anteroposterior and oblique plane images in order to automatically merge them with the preoperative CT. The next step involved positioning a compact robotic manipulator (400 g, 9 cm tall, 5 cm diameter) onto the bone-mounted platform, ensuring that it was precisely aligned with the planned trajectory, following the surgeon’s instructions (Fig. 1D). After tapping the screw paths with a thread tap through the expanded channels, the screw was manually inserted following the guide wire. The rods were percutaneously inserted from the upper side to the lower side, with the assistance of a screw extender. All patients underwent posterior long-segment fixation, and pedicle screws were placed 2 levels above and below the fractured vertebrae/disc. When the fracture type is transdiscal, pedicle screws were placed in the intact vertebrae above and below the fractured disc. When the fracture type is transvertebral, whether the fractured vertebra is fixed using screws depends on whether the pedicle is fractured. If the pedicle is not fractured, the fractured vertebra will be fixed (Fig. 2); otherwise, if the pedicle is fractured, it will be skipped and pedicle screws will be placed 2 levels above the fractured vertebrae (Fig. 1).

### Clinical evaluation

Follow-up evaluations were conducted at 1, 3, 6, and 12-month intervals after the surgical procedure, with additional yearly assessments if necessary. The effectiveness of the treatment was assessed using the visual analogue scale (VAS) for quantifying back pain severity, the Oswestry Disability Index (ODI) for evaluating disability, and the modified MacNab score to determine postoperative results during the most recent follow-up period. Neurological status was evaluated using the ASIA classification system. The collection of complications was conducted during both the intraoperative and postoperative periods.

### Radiographical assessment

Radiographic assessment included the evaluation of the sagittal Cobb angle, bone fusion status and pedicle screw placement. The sagittal Cobb angle is used to assess the achieved operative correction. It is defined as the angular measurement between a line parallel to the superior end plate of the vertebra located above the fracture and another line parallel to the inferior end plate of the vertebra positioned one level below the fracture [17]. Fracture healing is defined as the blurring of the fracture line on radiographs, the formation of bridging bone (Fig. 2), or the appearance of a trabecular pattern across the fracture site on CT imaging [12, 18]. The placement of the pedicle screws was assessed by utilizing axial CT scans and categorized according to the grading system of Gertzbein and Robbins [19]. The criteria for implant failure include screw breakage, screw pullout, peri-implant loosening, and rod breakage.

### Statistical analysis

Qualitative variables were presented using numerical values and percentages, while quantitative variables were expressed as the mean ± standard deviation or median. Paired sample t-tests were utilized to compare preoperative and postoperative measurements, with statistical significance defined as *P* < 0.05. The statistical analyses were conducted using IBM SPSS Statistics 27.0 software.

## Results

### Surgical results

All patients, including 11 males and one female, underwent posterior robotic-assisted percutaneous fixation. The average age was 62.8 ± 13.0 years, and the average duration of postoperative follow-up was 32.7 ± 18.9 months. The mechanism of injury was identified as low energy or no trauma history in 83% (*n* = 10), while a high energy injury was noted in 17% (*n* = 2). Fractures classified by the AO classification were B2 in 25% (*n* = 3) and B3 in 75% (*n* = 9) patients. Delayed diagnosis was present in 58% (*n* = 7) of patients, including 1 (14%) patient who experienced neurologic deterioration and 3 (43%) patients with secondary pseudarthrosis. Delay of surgery (> 72 h) occurred in 8 (67%) patients. The mean operative time was 119.6 ± 32.2 min, and the median blood loss was 50 (50, 250) ml. The average change in hemoglobin concentration before and after surgery was 1.6 ± 8.6 g/dl. Mean hospital stay duration was 15.0 ± 8.0 days. One patient experienced delayed neurologic deficit after surgery, resulting in a change of grade from ASIA E to ASIA C. The patient underwent emergency spinal canal decompression with the assistance of a microscope, as it was determined that the cause was compression caused by a hematoma, and the neurological status returned to normal after 2 weeks. The remaining patients did not experience any postoperative complications, and there were no deaths during the follow-up period (Table 2).

**Table 2:** Surgical treatment and outcomes

Case	Internal stabilization	Operation time (min)	Hemorrhage (ml)	Perioperative complications	Death during FU	Length of hospital stay (day)	Time of bony union (mon)
1	T10-L4	180	400	No	No	14	7
2	T3-8	180	300	No	No	26	12
3	T12-L3	130	50	No	No	11	4
4	L1-5	120	300	No	No	12	6
5	T9-12	100	100	No	No	20	8
6	T10-L2	110	50	Yes (neurological deficit)	No	32	7
7	T10-L2	95	100	No	No	14	6
8	T12-L3	120	50	No	No	7	5
9	T9-T12	90	30	No	No	6	6
10	T12-L4	130	40	No	No	7	6
11	T12-L4	100	50	No	No	12	5
12	T9-L1	80	50	No	No	19	6

L, lumbar; T, thoracic; FU, follow-up

### Clinical results

All patients expressed satisfaction with the outcome of the surgery and reported a reduction in their back discomfort. The preoperative VAS value showed a significant improvement, decreasing from 6.8 ± 0.9 to 1.3 ± 1.0 the final follow-up (*P* < 0.05). Similarly, the ODI value demonstrated a remarkable enhancement, reducing from 83.6 ± 6.1% before surgery to 11.8 ± 6.6% at the most recent follow-up (*P* < 0.05) (Table 3). Based on the modified Macnab criteria, clinical efficacy was assessed as excellent in 10 cases and good in 2 cases during the most recent follow-up evaluation. One patient who had ASIA D neurologic deficit before surgery improved to ASIA E, and the internal fixation was removed 2 years after the operation.

**Table 3:** Preoperative and last follow-up patient data

	Preoperative	Last follow-up	*P* value
VAS	6.8 ± 0.9	1.3 ± 1.0	< 0.001
ODI (%)	83.6 ± 6.1	11.8 ± 6.6	< 0.001
Cobb angle (°)	15.2 ± 11.0	8.3 ± 7.1	0.002

VAS, visual analogue scale; ODI, oswestry disability index

### Radiologic findings

**Fig. 3:**
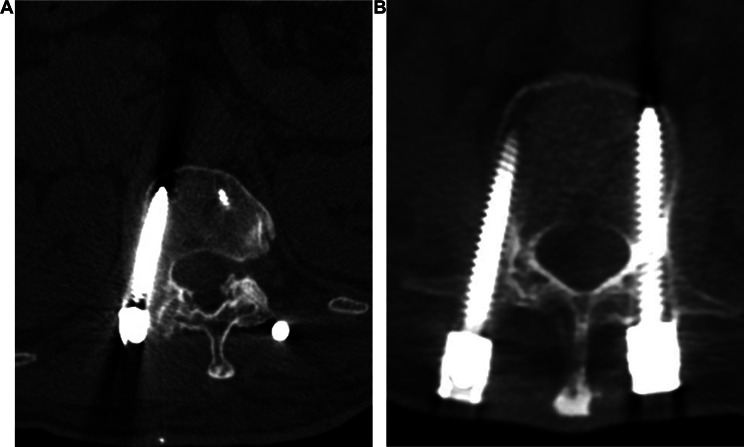
Postoperative computed tomography (**A**-**B**) revealed that the screws were positioned outside the lateral wall of the pedicle

All patients achieved successful fracture healing, with an average healing time of 6 (5.3, 7.0) months, and no patient experienced implant failure. Out of the 108 screws implanted, 2 (1.9%) were improperly positioned, and both screws were located outside the lateral wall of the pedicle (Fig. 3). One patient did not have any clinical manifestations, so we did not perform further revision. Another patient experienced delayed neurologic deficit one day after surgery and underwent emergency spinal canal decompression. Although the cause was determined to be compression caused by a hematoma, we still repositioned the screw. The Cobb angle changed from 15.2 ± 11.0 preoperatively to 8.3 ± 7.1 at the final follow-up (*P* < 0.05) (Table 3).

## Discussion

Spinal fractures can occur in patients with AS even under low-energy impact, predominantly resulting in instability and involving all three columns of the spine, which presents a challenge for surgeons in terms of treatment [2, 4, 5]. However, fractures such as these may frequently go undetected on plain radiography and be masked by common symptoms in AS, leading to a delay in diagnosis [5]. It has been reported that delayed diagnosis was observed in 17.1–65.4% of cases with AS vertebral fractures [5, 20], and one-third of patients developed neurologic deficits [2]. Furthermore, this delay in diagnosis may result in non-union of the fracture, leading to thoracolumbar pseudarthrosis [21]. In our series, delayed diagnosis was present in 58% (*n* = 7) of patients, including 1 (14%) patient who experienced neurologic deterioration and 3 (43%) patients with secondary pseudarthrosis. The findings were consistent with those of previous studies. As previous studies have shown, there was a presence of both doctor’s delay and patient’s delay in the diagnostic process [5]. None of our patients experienced a delay in diagnosis due to the physician, which may be because we have been aware of the concealment and harmfulness with these spinal fractures and remained vigilant. However, there is still a need for improvement in the understanding of AS combined with spinal fractures. Especially for patients themselves, it is necessary to promptly seek medical advice when the nature of pain changes.

The effectiveness of conservative treatment is limited and often results in suboptimal outcomes, which may include the use of braces, rest, and anti-inflammatory drugs [22–24]. Due to the frequent occurrence of unstable injuries and a higher probability of encountering neurological symptoms, internal fixation is often considered necessary. The study conducted by Robinson et al. [25] demonstrated that surgical treatment could significantly improve patients’ survival rates. Additionally, Westerveld et al. [26] discovered that surgical treatment can enhance patients’ neurological function to some extent. Therefore, we recommend surgery in the absence of any surgical contraindications. The traditional posterior open surgery is a classic treatment method that also yields favorable clinical outcomes [27]. However, the surgical trauma is significant and there are more postoperative complications [9]. Nugent et al. [28] and Nakao et al. [22] reported surgical complications with incidences of 64% and 30.8%, respectively.

In recent years, minimally invasive surgery has been recognized by the majority of doctors, especially for elderly AS patients with more underlying diseases. It can achieve better clinical results and reduce the occurrence of surgical complications [9, 10, 29, 30]. Ye et al. [9] demonstrated that minimally invasive surgery can achieve effects similar to those of traditional open surgery, while reducing bleeding, trauma, and postoperative complications. Additionally, bone cement was used to enhance the screw holding force and prevent screw loosening in patients with severe osteoporosis [12, 31]. However, in patients with AS, the significant alterations in bony structure and anatomy result in a lack of identifiable landmarks, especially in the upper thoracic spine [32]. Therefore, acquiring high-quality intra-operative images can present challenges and impose rigorous technical demands on the operator, especially in minimally invasive surgery [9]. Bredin et al. [31] used a percutaneous technique to implant 228 pedicle screws, 6 (2.6%) of which were poorly positioned, including 1 within the spinal canal. According to a meta-analysis, Tian et al. [33] found that the incidence of screw malposition ranged from 10 to 31% when conventional techniques were used for pedicle screw insertion. In our series, the pedicle screw was implanted with the assistance of a robot and the accuracy rate was 98.1%, which surpasses the outcomes documented in previous studies [31, 33]. This finding also demonstrates the benefits of robot-assisted screw placement precision, thereby enhancing the safety of surgical treatment for patients with AS combined with thoracolumbar fractures. However, two pedicle screws remained positioned outside the lateral wall of the pedicle, and one underwent revision. The possible reason could be that the robot is not securely fixed, leading to guide pin slippage upon insertion due to bone sclerosis. Therefore, based on our experiential learning, we recommend slowly inserting the guide pin into the bone upon contact with the bone surface at maximum rotation speed. Fluoroscopy should be performed again after all screws placement to evaluate the position of the screw.

The repeated use of intraoperative fluoroscopy is essential for achieving more precise screw positioning, particularly in minimally invasive procedures performed on patients with AS [9]. Brooks et al. [34] demonstrated that minimally invasive surgery significantly increased intraoperative radiation exposure compared to traditional open surgery. Additionally, Kai et al. [11] identified radiation exposure as the only drawback of minimally invasive surgery in AS patients due to the increased difficulty in obtaining high-quality intraoperative images in the spine. The latest findings also indicate that prolonged exposure to low levels of radiation significantly increases the risk of mortality from solid tumors, causes damage to DNA and death of leukocytes, and has been classified as a “known human carcinogen” by the World Health Organization [35, 36]. Therefore, it is necessary to enhance occupational protection measures and reduce medical radiation exposure. In our study, the use of robot-assisted screw placement eliminates the need for repeated fluoroscopy during surgery, thereby significantly reducing radiation exposure. This finding has also been observed in other studies [13, 15, 37]. In the future, we will continue to closely monitor intraoperative radiation exposure, further present radiation-related data, and conduct controlled studies.

The clinical outcomes of our robot-assisted percutaneous fixation technique are consistent with previous studies on minimally invasive surgery, demonstrating consistently favorable results [12, 30, 31]. The VAS and ODI values were 1.3 ± 1.0 and 11.8 ± 6.6%, respectively, at the most recent follow-up. All patients successfully achieved union of the fracture, and there were no occurrences of implant failure. This is probably because we applied a robot-assisted technique to plan the trajectory of the screw and select a larger-sized screw in advance, achieving maximum fixation strength while maintaining the structural integrity of the vertebral pedicles [38]. Barkay et al. [39] demonstrated that postponing surgical intervention (> 72 h) in elderly patients with spinal ankylosing disorders may lead to an increase in medical complications and mortality. In our study, surgery was delayed in 8 (67%) patients; however, no patients died, and the incidence of postoperative complications was 8.3%, which was lower than that reported in other studies [12, 30, 39]. This could potentially be attributed to the limited sample size of participants in our research or the use of robot-assisted technology, which improves work efficiency and promotes early rehabilitation. One patient experienced delayed neurological deficits due to hematoma compression after surgery; fortunately, the neurological status returned to normal following spinal canal decompression surgery. From this, it can be inferred that robot-assisted surgery has the potential to enhance operational efficiency and mitigate complications. However, it is imperative not to disregard the limitations of minimally invasive surgery in terms of detecting deep tissue hemorrhage and ensuring prompt and effective hemostasis.

There were several limitations in our study. First, it was a retrospective study. Secondly, the study’s limited sample size emphasizes the need for future research with larger sample sizes to replicate these findings. Thirdly, we did not present data such as intraoperative fluoroscopy and specific screw size. Controlled studies with large samples are needed to further clarify the advantages of robot-assisted technology in the treatment of thoracolumbar fractures with AS. Finally, the relatively high cost of robot-assisted treatment necessitates a comprehensive evaluation of the need for such technology.

## Conclusions

Posterior robotic-assisted percutaneous internal fixation can achieve satisfactory outcomes for thoracolumbar fractures in patients with AS. However, while robot-assisted pedicle screw placement can enhance the accuracy of pedicle screw insertion, it should not be relied upon solely.

## Data Availability

The datasets used and analysed during the current study are available from the corresponding author on reasonable request.

## Abbreviations

CT: Computed tomography
AS: Ankylosing spondylitis
VAS: Visual analogue scale
ODI: Oswestry disability index
ASIA: American Spinal Injury Association
